# No Place for Predators? Time and again, advancing civilization has set people against large carnivores. On the front lines of Washington State, wildlife biologists hope that knowledge can trump fear, and ultimately lead to détente.

**DOI:** 10.1371/journal.pbio.0060040

**Published:** 2008-02-12

**Authors:** Liza Gross

## Abstract

When voters in Washington State banned hound hunting with an eye to protecting cougars, they unwittingly triggered a chain of events that wildlife biologists are still trying to understand.

*“The whole continent was one of continuing dismal wilderness, the haunt of wolves and bears and more savage men. Now the forests are removed, the land covered with fields of corn, orchards bending with fruit and the magnificent habitations of rational and civilized people.”—John Adams, 1756 [1]*.

Braced for savagery and sacrifice, European settlers in the New World came to the Pacific Northwest to tame the final frontier, the last refuge of “dismal wilderness.” While colonists in the East were poisoning, shooting, and trapping cougars to extinction during the 1880s, hundreds of thousands of pioneers flooded into what would soon become the new state of Washington. Following the well-worn pioneer playbook, Pacific Northwest immigrants converted forests to farmland and pasture and, fearing local predators as unacceptable threats to life, property, and game, paid bounty hunters to destroy all carnivores, large and small.

It would take over 30 years to exterminate the wolf, and several more decades to nearly eliminate the cougar—whose famously reclusive, solitary nature may have helped the cat survive a systematic eradication effort. Yet even against a backdrop of ongoing persecution, complaints of cougar attacks on livestock and game continued apace, and legislators, assuming that more complaints meant more predators, increased incentives for hunters to thin the population. By 1940, two United States Fish and Wildlife Service senior biologists reported that cougars had been “exterminated in practically all of their former range in the United States and are fast being eradicated from many parts of the West” [2].

Ironically, a measure passed to protect wildlife triggered a chain of events that led to the highest rates of human-caused cougar mortality since the height of the bounty era—even as the public clamored for higher cougar harvests.

Genetic evidence suggests that cougars evolved as a distinct species, Puma concolor, about 400,000 years ago, went extinct in North America during the last ice age, 10,000–12,000 years ago, then recolonized the continent from surviving populations in Central and South America [3], acquiring regional monikers along the way: catamount, panther, or painter east of the Mississippi; mountain lion in the Rocky Mountains and California; puma in the Southwest and Mexico; and cougar in the Pacific Northwest (see Figure 1). Once distributed more widely than any other American carnivore, P. concolor lost two-thirds of its historic range during the bounty era, which ended in most western states in the 1960s. Though cougar abundance increased in the West after states reclassified the cats as game animals, a designation that afforded them limited protection, the World Conservation Union considers P. concolor “near threatened” and warns that the species may soon qualify as “vulnerable” if current persecution and habitat degradation trends continue [4].

**Figure 1:**
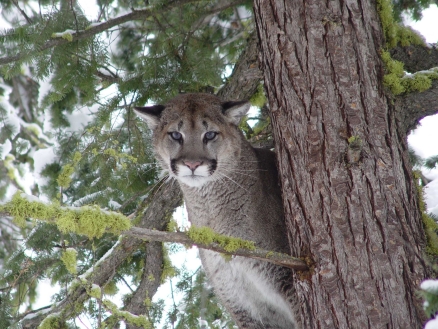
Echoes of the Past: Cougars Face the Same Threats Today That Nearly Eliminated the Species 100 Years Ago The cougar, Puma concolor, once the most widely distributed carnivore in the United States, was extirpated east of the Mississippi River by the early 1900s, save for a remnant population in Florida, which now struggles to survive with fewer than 90 individuals. Habitat destruction and persecution, the same forces that eradicated the cat from the East, continue to threaten the existing populations in the West. (Rich Beausoleil, WDFW)

Although reliable population estimates are notoriously difficult to generate for shy, wide-ranging, low-density animals, the Washington Department of Fish and Wildlife (WDFW) estimates that 2,500 to 4,000 cougars now inhabit the state—and they're finding it harder and harder to steer clear of humans. Washington's human population increased 21% between 1990 and 2000, far faster than the national average of 13% [5], leading to the destruction of over 70,000 acres of wildlife habitat each year [6].

Worried about the loss of prime cougar habitat and persecution of a top predator, animal welfare and environmental groups sponsored a statewide initiative (I-655) to outlaw the use of hounds to hunt cougars, a longstanding rural tradition—and the most efficient method of killing cougars—regarded as cruel by many city-dwelling voters. Anxiety over cougars, always a hot-button issue in the sparsely populated counties in northeastern Washington, reached a fever pitch after I-655 passed in 1996.

Yet in spite of predictions that an exploding cougar population would leave a trail of mutilated horses, dogs, and children, the measure's impacts were neither what supporters had hoped nor opponents feared. Ironically, it triggered a chain of events that led to the highest rates of human-caused cougar mortality since the height of the bounty era—even as the public clamored for higher cougar harvests. Wildlife biologists are still trying to understand the impacts of such heavy hunting on the ecology, behavior, and persistence of one of Earth's most secretive species. Whether they can find a way to help Washington residents and cougars coexist remains to be seen.

## Signs of Trouble

Catherine Lambert originally set out in 2002 to study regional variations in the reproductive response of cougars in the Pacific Northwest, but shifted gears when her radio-collared research subjects kept turning up dead. Lambert, then a student at Washington State University's Large Carnivore Conservation Laboratory, was shocked to find that nearly half of 52 radio-collared cats had been shot by hunters or in response to livestock depredation attributed to cougars. The mounting body count suggested that the population could be in serious trouble from overhunting. But Lambert's field observations ran directly counter to the popular belief that I-655 had triggered a cougar population explosion.

Contrary to popular belief—and the rationale behind legislation authorizing emergency and public safety hunts—increased complaints did not signal a growing cougar population.

That was because WDFW officials, well aware that losing hound hunting could reduce the number of cats killed, had liberalized hunting regulations to maintain traditional harvest levels. The agency extended the hunting season by six months, doubled the legal bag limit, and rolled cougar “tags,” or licenses, into big-game packages, which made them far more attractive to elk and deer hunters, known as “boot hunters.” Before the ban, WDFW sold 1,000 cougar tags a year. The new policy gave tens of thousands of deer and elk hunters the legal right to shoot cougars.

But perceptions die hard. Despite the agency's efforts—and even though I-655 allowed the agency to use hounds to protect the public—the incidence of cougar complaints, which averaged about 250 a year before I-655, more than doubled the following year before peaking at 936 in 2000. Cougar–human conflicts increased along with public anxiety, particularly in Okanogan County, an area where apprehension about cougars runs deep—the state's only recorded fatal attack on a human occurred here in 1924. The mood is captured by a 2003 column by Joel Kretz in a crusading libertarian monthly published across the border, *The Idaho Observer*, bearing the horror movie headline “Cougar Carnage at the Promised Land Ranch” and featuring a grisly photo of a wounded colt [7].

As frustration with the hound-hunting ban festered, Okanogan County commissioners threatened to defy state law by declaring open season on the “growing” cougar population, and by 2004, nine statewide bills had been introduced to reverse or circumvent I-655; two passed. Speaking for the Okanogan Farm Bureau, Kretz testified in favor of one that authorized hounds for public safety hunts and another that sanctioned emergency safety hunts in a pilot program that gave commissioners in five northeastern counties local control over cougar management. After years of complaining to politicians and the press about an “exploding” cougar population, Kretz was elected state representative from four of the five pilot counties in 2004. In 2007, he introduced a bill to extend the emergency safety hunt program another year.

As the “cougar problem” was debated on editorial pages, in public forums, and at state and county hearings—and the management of an enigmatic species moved from the hands of wildlife biologists first to voters and then to politicians—58,000 deer and elk hunters hit the woods with cougar tags in their pockets. And Catherine Lambert, worried about losing so many collared cougars, set out to test her suspicion that public perception about an exploding cougar population was dead wrong.

## Counting Cats

When Lambert joined the Large Carnivore Conservation Laboratory in 2002, its director, Rob Wielgus, was investigating declines of mule deer and endangered mountain caribou in the Selkirk Mountains, their last stand in the lower 48 states. Years of intensive timber harvest had transformed the ancient stands of old-growth forests rich with arboreal lichen, the mainstay of the caribou winter diet, into clear-cut blocks rife with forest edges and early seral vegetation like seedlings, saplings, and immature trees, destroying critical caribou habitat and forage.

White-tailed deer—historically rare in these parts—thrive on the immature vegetation left behind by forestry practices, and their numbers rose as those of native prey species declined. One explanation for the white-tails' success could be that they outcompeted native species for resources. But Wielgus found support for an alternative hypothesis called apparent competition, a negative interaction between prey species that occurs due to shared enemies rather than shared resources. As the white-tails invaded native ungulate range, moving upland in the summer, cougars followed, and their numbers expanded along with their prey base—for a while. Cougar predation on white-tails was density-dependent—it increased or decreased in relation to population growth—but continued to increase on caribou and mule deer even as their populations declined [8,9]. This trend, known as inverse density-dependent predation, signals that a species may be headed for extirpation.

Without intervention—such as culling cougars or changing forestry practices to discourage white-tails—Wielgus feared that neither the Selkirk caribou nor the mule deer populations would recover. Wildlife managers in the region agreed to increase hunting to limit predation. But why were cougars selecting for the native species? At first, Wielgus wondered if mule deer had hybridized with white-tailed deer and were somehow easier to kill, but DNA analysis showed no white-tail gene introgression into mule deer taken by cougars. Then his team analyzed “a huge dataset” on deer kills from global positioning system readings and realized that male cougars were killing white-tails at lower elevations, while females were killing mule deer at higher elevations.

Cougars appeared to be causing consternation everywhere, eating endangered caribou and deer and attacking livestock and pets, and even the occasional human. Cougar attacks on humans are extremely rare—lightning strikes are more common—but eight of nine documented attacks occurred in the 1990s, including two serious attacks on children in northeastern Washington, providing fodder for the post-I-655 legislative blitz to expand hunting. What if all the problems were the result of a hunted—rather than a growing—cougar population? Evidence from studies on African lions [10] and wolves [11] suggests that heavy hunting reduces the average age in carnivore populations—and a survey of cougar attacks on humans over the past century (1890–1990) found that most attacks involved yearlings [12,13]. Maybe the problem wasn't too many cougars, but too many unruly juveniles.

When Lambert began work on Wielgus's cougar project, his team had already started to capture and radio-collar 52 cats, first in study sites around the Selkirk Mountains in northeastern Washington, southern British Columbia, and northern Idaho, and later in another site in Colville National Forest in northeastern Washington (see Figure 2). The team measured annual maternity rates and survival rates of kittens and radio-collared adults and plugged the numbers into a population viability model to estimate projected growth rates. The results were sobering. The average survival rate for females was 77%, but nearly 40% of kittens and yearlings were dying each year—and nearly 70% of adult males.

**Figure 2:**
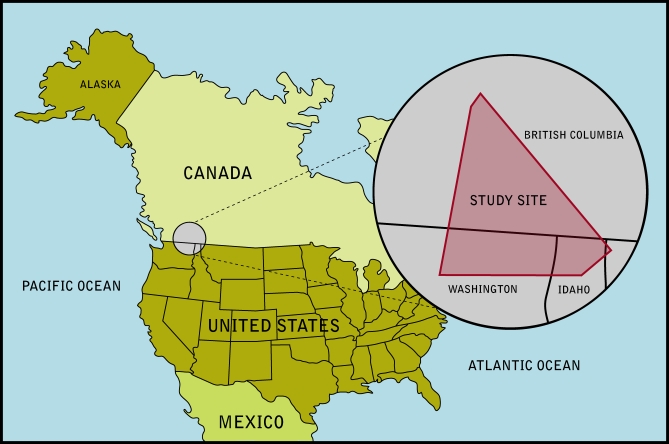
Studying Cougar Population Dynamics and Viability in the Selkirk Mountains Researchers with the Large Carnivore Conservation Laboratory at Washington State University captured and radio-collared 52 cougars living in the Selkirk Mountains (in northeastern Washington, northern Idaho, and southeastern British Columbia) and Colville National Forest (in northeastern Washington) to test the hypothesis that a growing cougar population was responsible for increased cougar–human conflicts. They found that heavy hunting of the population, implemented in part to reduce conflicts, may have actually exacerbated the problem.

“This is where it gets really depressing,” Wielgus told a recent national meeting of science writers, where he presented his latest results. Aside from some older females, “we don't have any four-year-old cougars left.” Hunters, as Lambert discovered firsthand, accounted for 92% of cougar deaths—and indirectly killed five of 21 dependent kittens by shooting their mothers.

Based on an annual census, the minimal total density fell from 1.46 cougars/100 km^2^ at the beginning of the study to 0.85 cougars/100 km^2^ in the last two years. As Lambert reported last year in the *Journal of Wildlife Management* [14], even the starting density was much lower than most other cougar populations (4.2 cougars/100 km^2^ in Alberta, Canada, for example, and 3.5–3.7 cougars/100 km^2^ in British Columbia).

The population was growing at the start of the study, when Wielgus discovered cougar selection on caribou and mule deer, but started to decline by 30% a year in 2000—just when complaints reached an all-time high. If current harvest rates continued, the cougar population would disappear within 30 years. Contrary to popular belief—and the rationale behind legislation authorizing emergency and public safety hunts—increased complaints did not signal a growing cougar population. “As complaints were going up, the population was tanking,” Wielgus says.

The intensive hunting in the Selkirks did achieve one thing: it relieved predation pressure on caribou and mule deer. Mule deer populations have recovered beyond expectation, but white-tailed deer are also increasing—at the rate of 30% a year. The strategy just facilitated the invasion of the white-tail, Wielgus says, which will likely outcompete mule deer for resources down the road.

## Cryptic Population Dynamics

Wielgus's team continued studying the population in Colville National Forest, another area with fears about a growing cougar population and heavy hunting, though on a smaller scale. Based on low survival and maternity rates—kitten survival rates were also low—the population was in rapid decline, mostly due to female mortality. Yet by census count, the population appeared stable over time, but not sustainable. The team found more juveniles than expected, no decline in total or adult density, and a shift in population structure toward younger independent males. The hunted population acted as a sink, attracting immigrants and younger animals, which masked the loss of females. But males won't stick around if there aren't any females left. And a population without females has no future.

Wielgus saw a similar dynamic with grizzly bears when he tested the notion that trophy hunting increases offspring production, survival, and population growth by reducing the abundance of competitive or cannibalistic adult males. Deer and other traditional game animals typically respond to predation (or hunting) with increased reproduction and survival. But top carnivores, which have not adapted to predation over evolutionary time, should not be expected to respond like prey species, Wielgus reasoned. Instead, he found that hunting older adult male grizzly bears in small populations attracted dispersing, potentially infanticidal males, led to increased sexual segregation and reduced reproduction, and ultimately compromised population growth and persistence. Reduced cub production, Wielgus argued, occurred because adult females moved into territories where resources and, presumably, infanticidal males were scarce [15].

The same thing appears to be happening with cougars. “As we kill all these big resident adult animals, the younger guys come to the funeral,” Wielgus says. And that could explain cougar selection on mule deer. The immigrant males hang out in lower elevations, killing white-tailed deer where prey densities are very high, but females move to higher elevations—where prey densities are lower—and kill mule deer incidentally. It stands to reason that females would go where prey densities are high, but they don't. Wielgus is testing the possibility that females go to resource-poor areas to avoid immigrant males—which easily travel 150 miles (240 km) to find a potential mate—to protect their kittens. He doesn't have direct evidence of infanticide, but notes that more kittens turn up dead when unrelated immigrant males enter the system than when their fathers are there.

Wielgus found the probable source population for the Colville immigrants in another study area to the southwest, where white-tailed deer are still rare. Unlike both heavily hunted populations, kitten and adult female survival rates were high—and adult male survival rates were twice as high. In this “lightly hunted” population, the mule deer population was healthy and cougar complaints were low. Hunting was acting as a form of habitat degradation. The lightly hunted populations have stable habitat use, home ranges, and population growth. But in heavily hunted populations, “we appear to have chaos,” Wielgus says, with no adult males, an influx of immigrants from surrounding areas, home ranges and densities “shifting all over the place,” more infanticide, and far more cougar–human conflicts. “And we suspect—this is what we're studying now—that these teenage males cause more problems than older residents and that this heavy harvest exacerbates the problem rather than making it better.”

## From Conflict to Coexistence

While state law prohibited WDFW officials from commenting publicly on I-655, agency biologists saw the two legislative measures authorizing hounds for safety hunts as an opportunity to reassert control of cougar management (see Box 1). For the five-county pilot program, for example, the agency incorporated a quota system, in which the harvest ends once either the female or total cougar quota is reached. It's a policy that Rich Beausoleil, a bear and cougar specialist with WDFW, wants to see implemented statewide. With the number of cougar tags sold increasing every year—over 66,000 were sold in 2007—a quota appears more critical than ever. For now, some counties without the quota still cull cougars to manage conflicts, based on the old game management plan.

**Box 1. Managing Good Intentions**

Ironically, once the initiative to ban hound hunting passed, “presumably to protect cougars,” Rob Wielgus says, “it resulted in a big harvest of cougars, a decline in the female component, and the influx of teenage males. The road to hell is paved with good intentions. All my data suggest that we should go back to hound hunting, which is regulatory, density-dependent, and sustainable.”

Whereas hound hunters typically select for trophy toms, deer and elk hunters kill indiscriminately. “Hunters are up in a tree stand waiting for a deer to come by and all of a sudden they see a cougar,” says WDFW's Rich Beausoleil. Before the big-game package was created, he says, “they wouldn't have had a license to take that cougar, but now, because they have the tag, they take it.”

Beausoleil and Donny Martorello, a WDFW carnivore expert, studied harvest statistics before and after the 1996 ban [21]. Before the ban, “you'd see a 60% male harvest consistent for 50 years, always 60/40,” Beausoleil says. But when the initiative passed, the trend flip-flopped, with up to 65% female harvest. “When you use dogs, you can tree a cougar and look up in the tree and say, ‘Well, that's a female or a young male, let's let this one go and we'll wait for a big tom,’ because everybody wants a big tom.” With hounds instead of deer and elk, or “boot,” hunters, he explains, “we could be protecting the female portion of the population.”

Wielgus thinks that hound hunting may lead to a self-regulated, density-dependent harvest, a theory he's testing now. Hound hunters typically go where residents have reported an incident, and tend not to overharvest, since cougars occur at such low densities. But deer and elk hunters kill far more cougars incidentally. “We documented that as cougar numbers go down, deer numbers go up, and what I think is happening is that when deer numbers go up, you get more boot hunters, which means more guys with cougar tags in their pocket, so they kill more cougars, which means more deer, which means they kill more cougars,” he explains. The smaller the cougar population, the higher the hunting. “That's inversely density-dependent,” Wielgus says. “Some people call that the road to extirpation.”

For Wielgus, mounting evidence over the past decade argues that it's time to rethink wildlife management models. “We're learning all kinds of things that are counterintuitive,” he says, like the notion that shooting animals may not reduce their numbers. Traditional management models have been based on white-tail populations in Pennsylvania and deer mice, he says, but large carnivore behavior and population dynamics are completely different.

As for understanding the dynamics of cougar predation on endangered prey, Wielgus says that it's important to ask why the predators are there in large numbers. “If there's a really high density of white-tails, the predators are going to come in, so the long-term solution is getting the habitat back in shape such that it's not so attractive to white-tailed deer,” he says. “And if white-tails are expanding into areas where they're historically nonnative and wolves aren't there to kill them, which they historically may have done elsewhere, something has to take their place.” Hunting may be the best short-term solution, Wielgus says. “You could say, ‘Let's not hunt the white-tails,’ but then it's bye-bye mountain caribou and bye-bye mule deer. Meanwhile our entire ecosystem is suffering.”

Heavy hunting, as Wielgus showed, alleviated predation pressure on endangered ungulates only by sending the cougar population to the brink of collapse. And heavy hunting on a smaller scale didn't even reduce the population, or the complaints, because of increased immigration. Since predatory behavior is learned—a cougar might discover that caribou herds concentrated in small forest patches or livestock in fenced pastures make easy pickings—individual cats can cause a lot of trouble (see Figure 3). When Wielgus's team studied cougar predation on endangered caribou, they discovered that although all 22 cougars lived near caribou habitat, only two spent significant time among caribou—and only one learned to kill them [16]. Rather than wide-scale hunting to reduce human conflicts, the aim of the bills passed after I-655, removing one problem cat is likely to prove far more effective.

**Figure 3:**
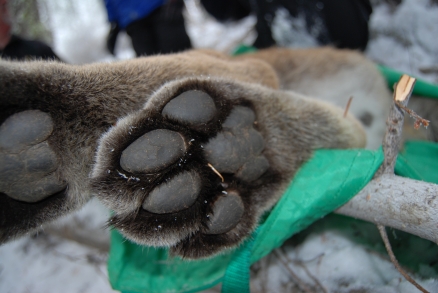
A Cougar Paw Cougars tend to keep their claws retracted when not in use, which is why their tracks leave no claw marks. A large male kills on average one deer or elk every week to 12 days and may stalk its prey for an hour or more, then attack with a sudden burst of speed, aiming for a quick kill by breaking the animal's neck. (Rich Beausoleil, WDFW)

WDFW is now seeking public comment on its next game-management plan [17], which will drive the department's management actions for the next six years. Beausoleil says that state and university wildlife biologists have collected so much data on the population dynamics of hunted populations over the past decade that the agency now has a clearer understanding of how to manage, and protect, the population—based on rigorous science, not perception.

For the wildlife officials who spend their days mediating conflicts with cougars, the prospects for coexistence depend on public education. “If you put your dog out to do his business at 1 a.m. with no lights on and no noise, and a cougar just happens to be passing by, it's likely to figure out that lunch comes out the back door every night at one in the morning,” says WDFW enforcement officer Jim Brown.

Convincing the public to accept top predators as an integral part of a healthy landscape is Beausoleil's long-term goal. But it won't be easy. Gray wolves have been sighted around Lake Chelan, just west of Okanogan County, far from established packs in neighboring Idaho. “If the wolves get here, we won't even be thinking about cougars,” says State Representative Kretz. “They're a hundred times worse.”

Hunting was acting as a form of habitat degradation. The lightly hunted populations have stable habitat use, home ranges, and population growth. But in heavily hunted populations, chaos prevails.

When top predators like cougars and wolves disappear, surprising things happen. By creating a “landscape of fear,” predators change prey behavior. Reintroducing gray wolves into Yellowstone National Park drove deer, elk, and moose out of willow stands, releasing grazing pressure on songbird habitat and increasing songbird diversity [18]. The absence of cougars and wolves in the eastern United States has been blamed for songbird declines there. Both top predators typically snack on raccoons, foxes, and skunks, which in turn favor the eggs of warblers and other songbirds. Without predation pressure from cougars and wolves, mid-sized predator populations exploded and destroyed the reproductive capacity of songbirds [19].

Such benefits are a tough sell among those who view large carnivores as threats to life and property. While public safety is still a top priority for WDFW, agency biologists, unlike politicians, must also worry about the needs of resident wildlife. “One of the things we'll never get a handle on is the folks who move to the end of a box canyon in the middle of nowhere, and maybe they come from the city, and they see a cougar and say, ‘Hey, I saw a cougar, you've got to remove him,’ ” says Beausoleil. “Well, no, that's not what we do. You're living in cougar country now.” He hopes that one day the developers whose brochures tout all the bounding hills, wildflowers, deer, and elk will tell people about all the bear and cougar there too.

In 1946, US Fish and Wildlife biologist Stanley Young wrote that cougars “are so destructive to man's interests that they cannot be tolerated except in the wildest areas” [2]. But he also thought that with “great stretches of wilderness that will probably never be touched by puma-control campaigns…the species will long continue to exist in America.” Arguing that predators must be destroyed to conserve game and livestock, Young's colleague and coauthor, E. A. Goldman, echoed that sentiment: “Large predatory animals destructive of livestock and game no longer have a place in our advancing civilization” [20].

The days when wildlife managers viewed the cat of many names as vermin to be eradicated are long gone. Modern managers promote predators' role as guardians of ecosystem integrity, but they are also employees of the state and must balance the needs of the species with the will of the electorate. As America's great stretches of wilderness rapidly disappear into the transfigured landscapes of advancing development, the fate of the cougar depends on whether “rational and civilized people” can see that the world would be a poorer place without predators.

## Abbreviations

WDFW: Washington Department of Fish and Wildlife
